# Dual-function quorum-sensing systems in bacterial pathogens and symbionts

**DOI:** 10.1371/journal.ppat.1008934

**Published:** 2020-10-29

**Authors:** Kelsey Barrasso, Samit Watve, Chelsea A. Simpson, Logan J. Geyman, Julia C. van Kessel, Wai-Leung Ng

**Affiliations:** 1 Department of Molecular Biology and Microbiology, Tufts University School of Medicine, Boston, Massachusetts, United States of America; 2 Program in Molecular Microbiology, Graduate School of Biomedical Sciences, Tufts University, Boston, Massachusetts, United States of America; 3 Department of Biology, Indiana University, Bloomington, Indiana, United States of America; Nanyang Technological University, SINGAPORE

## Introduction

Quorum-sensing (QS) systems, which rely on the production and detection of chemical signals called autoinducers (AIs) made by the bacteria themselves, are classically thought to be employed as a means to sense “self,” ensuring that bacteria cooperate and share resources to benefit their kin. Thus, most QS receptors are found to be specific for their cognate AIs. Although stringent signal specificity is considered fundamental to the fidelity of QS, receptors that respond broadly to non-self AIs have been identified. These “promiscuous” QS receptors are thought to function as interspecies signaling systems that are implicated in both competition and cooperation between microbes in polymicrobial communities [1,2].

Additional signal-sensing strategies have evolved for the QS systems in pathogenic and symbiotic bacteria which need to interact intimately with their hosts. Here, we discuss the organization and functions of QS circuits that harbor a dual-sensing function by detecting both endogenously produced AIs as well as chemical cues present inside the host. Co-opting the use of QS circuits to incorporate both microbial and host-derived information into their sensing repertoire allows proper spatial and temporal regulation of the expression of determinants critical for pathogenesis and symbiosis.

### AI-3/epinephrine sensing by EHEC QseC

**Fig 1:**
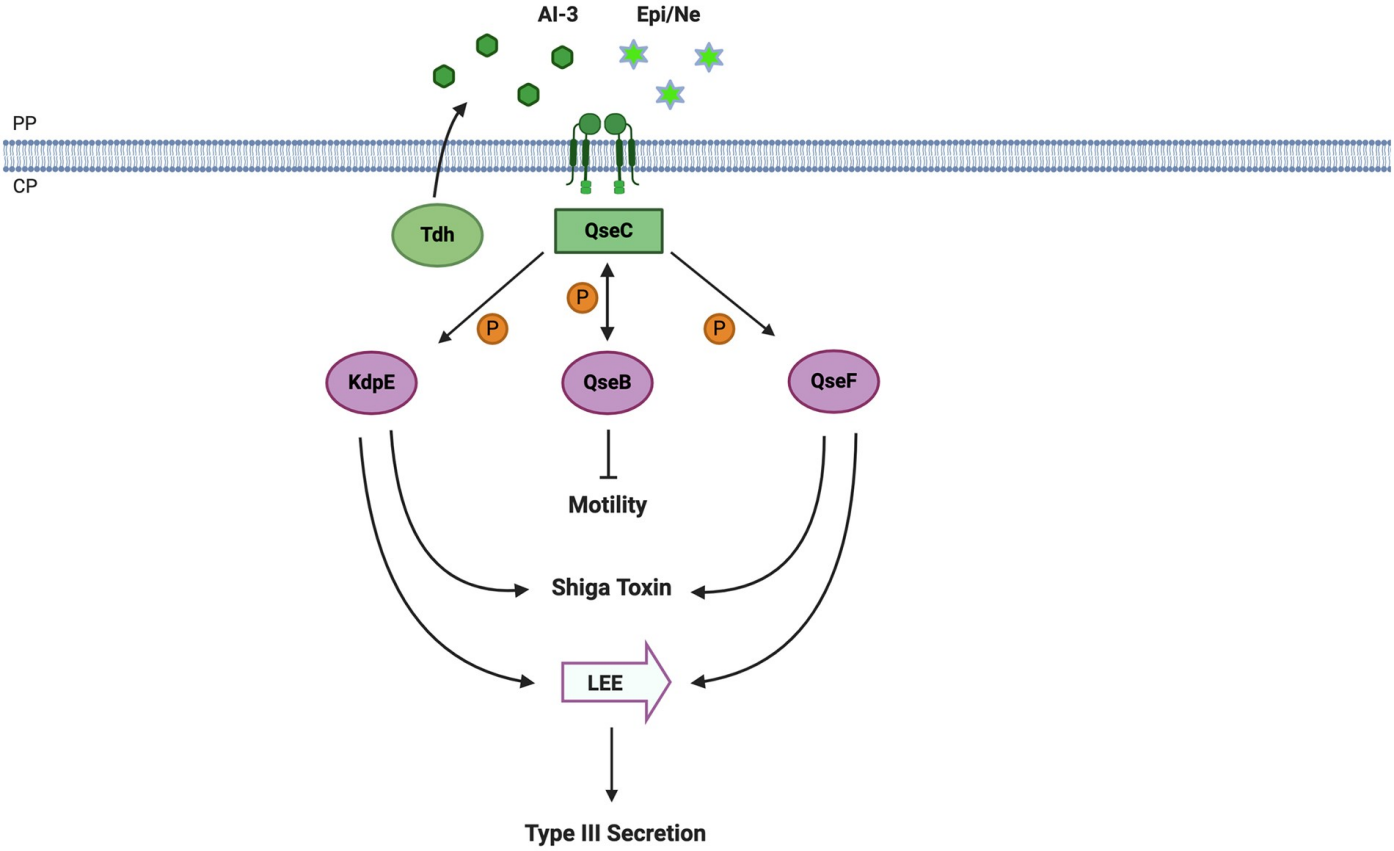
The AI-3/Epi/NE signaling pathway in EHEC. The HK QseC detects Epi and NE made by the host, as well as AI-3 produced by the EHEC enzyme TDH. Binding of these molecules to the HK enables QseC to control the activity of the 3 RRs KdpE, QseB, and QseF, which results in regulation of downstream virulence genes. AI-3, autoinducer-3; EHEC, enterohemorrhagic *Escherichia coli* O157:H7; Epi, epinephrine; HK, histidine kinase; NE, norepinephrine; RRs, response regulator; TDH, threonine dehydrogenase. Created with BioRender.com.

The QseC histidine kinase (HK) of enterohemorrhagic *Escherichia coli* O157:H7 (EHEC) is the earliest reported example of a QS receptor detecting both bacteria-made AIs and host-generated signals [3]. EHEC colonizes the human colon, and virulence is dependent on Shiga toxin, flagella/motility, and a type III secretion system (T3SS). Expression of the genes encoding these factors are all activated by QseC upon detection of various signals [4]. QseC is required for EHEC motility by modulating the phosphorylation state of its cognate response regulator (RR) QseB. Phosphorylated QseB binds to the regulatory region of *flhDC* (the master regulator of the flagellar regulon) (Fig 1) [5]. The dephosphorylation of QseB by QseC is critical to derepress *flhDC* and maintain motility gene expression, particularly since another HK PmrB also phosphorylates QseB [6,7]. QseC also phosphorylates 2 additional RRs QseF and KdpE, and together, these 2 RRs activate the expression of T3SS genes on a pathogenicity island called locus of enterocyte effacement (LEE) as well as the Shiga toxin gene *stx2* (Fig 1) [4,8,9].

The kinase activity of QseC is modulated by multiple signals (Fig 1). QseC is activated by a self-produced autoinducer AI-3, which consists of a group of molecules belonging to the pyrazinone family, whose biosynthesis depends on threonine dehydrogenase (TDH) [10]. Synthetic AI-3 compounds added to EHEC cells activate virulence gene expression through QseC with varying potencies and specificities; however, a direct ligand binding interaction between AI-3 and QseC has not been demonstrated [10]. QseC also separately detects human adrenergic hormones epinephrine (Epi) and norepinephrine (NE) [3,4,11]. Epi/NE directly activates the kinase activity of QseC in vitro [11] and QseC-dependent virulence gene expression in EHEC [4]. The in vivo role of QseC sensing of Epi/NE in host colonization was studied using *Citrobacter rodentium* carrying a LEE island similar to that from EHEC [12]. *C*. *rodentium* is deficient for colonizing dopamine β-hydroxylase knockout (Dbh^−/−^) mice, which do not produce Epi/NE. Similarly, *qseC* null mutants were also impaired for colonizing the mouse intestine, highlighting the importance of host signal sensing during host colonization [12]. Overall, these studies have established that QseC acts a crucial link integrating both host-derived signals (Epi and NE) and self-produced AI molecules (AI-3). It should also be noted that the exact regulatory mechanisms of QseC on target gene regulation are diverse among different *E*. *coli* subtypes. For example, in uropathogenic *E*. *coli* (UPEC), QseB phosphorylation state can be cross-regulated by another HK PmrB in response to iron to confer polymyxin resistance [7,13,14]. While the role of Epi/NE sensing may not be universal among different *E*. *coli* strains and subtypes, QseC signaling has been shown to be critical for virulence in many strains of enteric pathogens such as *Salmonella*, UPEC, and enteroaggregative *E*. *coli* (EAEC) [4,15,16].

### *Vibrio* QS systems that detect host-generated signals

**Fig 2:**
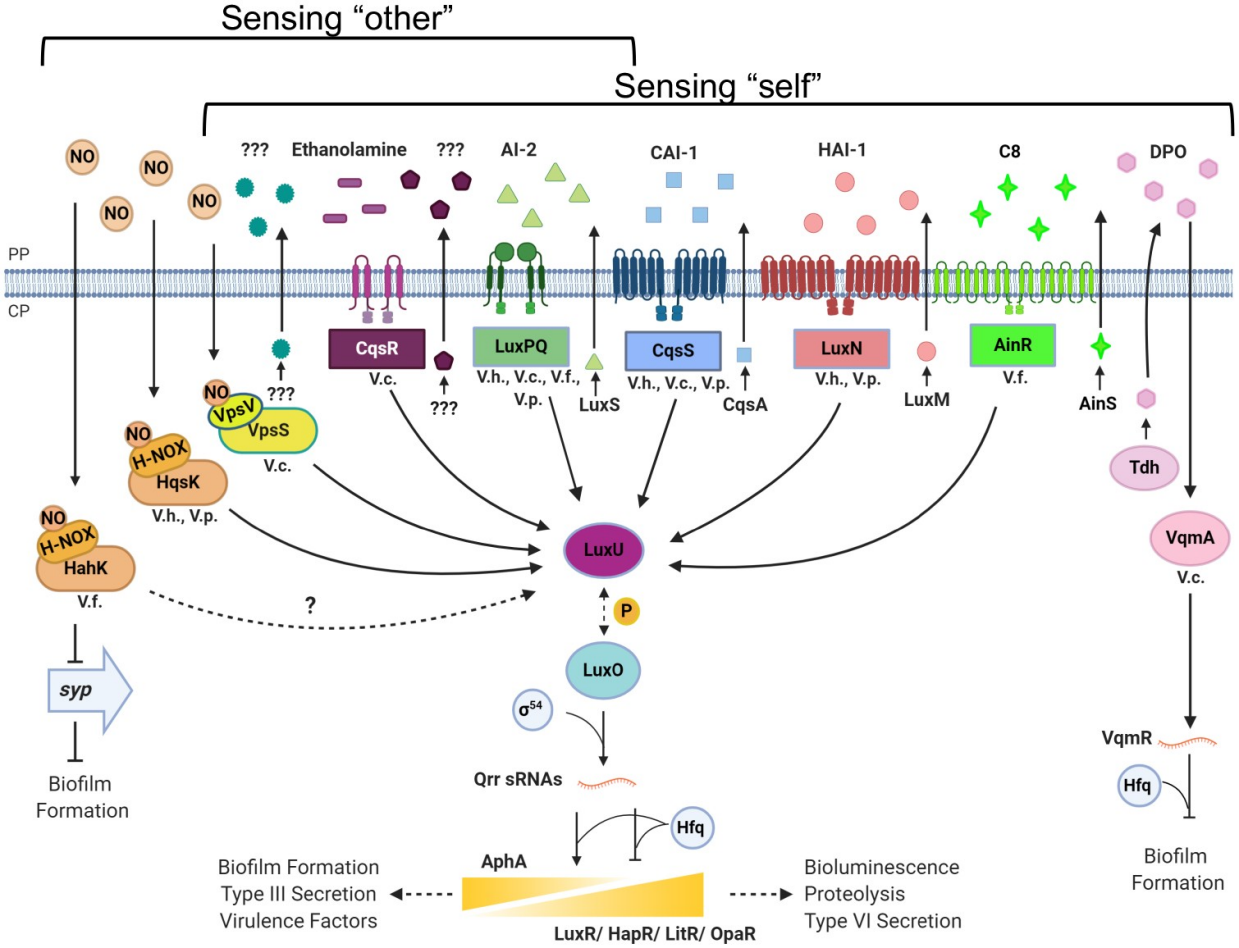
*Vibrio* signaling pathways for QS and host sensing. Various HK receptors in *Vibrio* species recognize distinct signals produced by the bacterium (sensing “self”) and/or produced by host cells or neighboring bacteria (sensing “other”). The conservation of the HKs varies among species, and this figure indicates the proteins and signals determined in the literature for each species (V.h, *Vibrio harveyi*; V.c., *Vibrio cholerae*; V.p., *Vibrio parahaemolyticus*; V.f., *Vibrio fischeri*). Signals produced by enzymes (if known) are indicated for each system. In the absence of cognate signals, phosphorylation (P) of LuxU and LuxO leads to production of AphA and low production of LuxR (V.h.)/HapR (V.c.)/OpaR (V.p.)/LitR (V.f.) and expression of biofilm, virulence, and type III secretion genes. In the presence of signals, dephosphorylation of LuxU drives production of LuxR/HapR/OpaR/LitR and expression of bioluminescence, proteases, and type VI secretion genes. The NO/H-NOX/HahK pathway in V.f. inhibits *syp* gene expression and biofilm formation. The VqmA/DPO pathway inhibits biofilm formation in V.c. AI-2, autoinducer-2; CAI-1, cholera autoinducer-1; CP, cytoplasm; DPO, 3,5-dimethyl-pyrazin-2-ol; HAI-1, harveyi autoinducer-1; HK, histidine kinase; H-NOX, heme nitric oxide/oxygen binding; NO, nitric oxide; PP, periplasm; QS, quorum sensing. Created with BioRender.com.

Many *Vibrio* species including *Vibrio cholerae*, *Vibrio harveyi*, and *Vibrio fischeri* spend part of their life cycle inside animal hosts either as a pathogen or as a symbiont. These species use multiple QS systems to regulate the expression of the genes involved in host colonization [17]. Emerging evidence suggests that *Vibrio* species, similar to EHEC, also integrate host-derived chemical cues to modulate their overall QS responses. To illustrate this idea, we first focus on the canonical QS circuit of *V*. *cholerae* composed of 4 HK receptors CqsS, LuxPQ, CqsR, and VpsS [18] (Fig 2). At low cell density (LCD), these 4 HKs function in parallel to phosphorylate RR LuxO through an intermediate phosphotransfer protein LuxU. Phosphorylated LuxO promotes and inhibits the production of master transcriptional regulators AphA and HapR, respectively, resulting in the activation of virulence and biofilm gene expression at LCD, which is critical for *V*. *cholerae* host colonization [18]. At high cell density (HCD), binding of the cognate signals to the receptors leads to kinase activity inhibition, resulting in dephosphorylation of LuxO and expression of HCD QS genes. Some of the AIs detected by these QS receptors are well characterized: CqsS and LuxPQ detect the *Vibrio*-specific signal CAI-1 (*S*-3-hydroxytridecan-4-one) and the “universal” signal AI-2 in its cyclic, borated form (*S*-2-methyl-2,3,3,4-tetrahydroxytetrahydrofuran-borate), respectively. AI-2 is made by many bacteria via the enzyme LuxS and is considered an interspecies signal [19]. The 2 additional *V*. *cholerae* QS receptors, CqsR and VpsS, have been demonstrated to respond to self-made chemicals present in spent culture media; however, the identities of these signals remain unknown [18] (Fig 2). Similar parallel circuit architecture is found in other *Vibrio* species; however, the receptors used for signal perception can be variable. For example, the LuxN HKs in *V*. *harveyi* and *Vibrio parahaemolyticus*, and AinR HK in *V*. *fischeri*, which are all absent in *V*. *cholerae*, detect acyl homoserine lactones (AHLs; Fig 2) [19] and are distinct from the cytosolic LuxR AHL receptor in *V*. *fischeri*. Here, we will discuss how 2 host-derived signals, ethanolamine and nitric oxide (NO), are detected and integrated into these parallel HK-based QS systems.

### Integration of ethanolamine sensing into the QS circuit

Ethanolamine is a common intestinal metabolite generated during host and bacteria membrane turnover. In an unbiased chemical screen, ethanolamine was found to specifically interact with the periplasmic ligand-binding domain of CqsR. In *V*. *cholerae* mutants expressing only CqsR but not the other 3 QS receptors, ethanolamine induces a premature HCD QS response to inhibit virulence gene expression and limit host colonization [20]. Yet, *V*. *cholerae* mutant defective in producing ethanolamine is still proficient in QS, suggesting ethanolamine functions only as an external cue for CqsR, and additional signals must be endogenously made by *V*. *cholerae* and detected by CqsR. While the exact physiological function of ethanolamine sensing by CqsR remains unclear, the ethanolamine concentration is notably higher in the large intestine than that in the small intestine [20], and therefore, ethanolamine could be used as a proxy for niche identification. Interestingly, previous studies have demonstrated that ethanolamine both positively and negatively affects host colonization and virulence during infection with other enteric pathogens [21,22].

### Integration of NO sensing into the QS circuit

NO is produced by a variety of animal cells as an antibacterial mechanism. Upon NO sensing, some bacteria express a set of nitrosative response genes to counteract this toxic compound [23]. Heme NO/O_2_ binding (H-NOX) proteins are a broadly conserved family of sensor proteins that bind NO within an Fe(II)-heme domain [24]. H-NOX modulates the activity of a HK called H-NOX-associated QS kinase (HqsK) encoded in the same operon as H-NOX [25–27]. In *V*. *harveyi* and *V*. *parahaemolyticus*, HqsK feeds into the parallel QS circuitry made of LuxPQ, CqsS, and LuxN described above (Fig 2). In the absence of NO, HqsK phosphorylates LuxO via LuxU. When NO is present, it binds to H-NOX, and this complex inhibits the kinase activity of HqsK. This decreases the pool of phosphorylated LuxU and LuxO, resulting in a premature HCD QS response (e.g., increase in light production) in *V*. *harveyi* [25,26]. In *V*. *fischeri*, H-NOX/NO inhibits the HqsK homolog, HahK (Fig 2), resulting in decreased biofilm formation via inhibition of *syp* transcription [28] and decreased expression of genes encoding hemin transport [29]. It is not yet clear if H-NOX influences HKs in the *V*. *fischeri* QS circuit, although LuxPQ and the downstream components are conserved in *V*. *fischeri* (Fig 2).

NosP proteins are another class of NO-sensing proteins that are widely conserved in bacteria and are also encoded in operons with cognate signaling proteins [30]. In *V*. *cholerae*, NosP (also called VpsV) binds NO and inhibits the autokinase activity of VpsS (encoded in the same operon) in vitro [30] (Fig 2). In this way, *V*. *cholerae* NosP bound to NO appears to function analogously to *V*. *harveyi* H-NOX to inhibit phosphorylation of LuxU and could potentially feed into the QS pathway. However, the exact physiological role of NO sensing by NosP/VpsV in *V*. *cholerae* QS gene regulation is unclear.

Because there are no identified NO synthase genes encoded in these *Vibrio* species, it is hypothesized that NO acts as an interkingdom signaling molecule between bacterium and host. For example, in *V*. *fischeri*, during early stages of colonization of the light organ in the bobtail squid *Euprymna scolopes*, the surface epithelium of the squid secretes mucus that contains NO [31]. *V*. *fischeri* cells first adhere to the mucus and form aggregates, and then the bacteria disperse and migrate through pores to eventually colonize the crypts of the light organ. NO inhibits aggregation and biofilm formation, and other signals such as calcium positively influence biofilm formation [28]. Thus, the integration of both NO and calcium signaling may balance aggregation and biofilm formation to a level that enables the bacteria to disperse from the aggregates to colonize the light organ. Additionally, the repression of hemin transport by HahK likely prepares the bacteria for the iron limited environment of the host light organ. In tandem, these results indicate that the *V*. *fischeri* bacteria rely on NO host signaling to colonize and adjust to the environment in the light organ.

### Concluding remarks and further perspectives

It is now clear that the bacterial QS response is not only regulated by self-made AIs, and the boundary between “self-sensing” and other signaling networks becomes blurry. Combining information about the environment together with QS, especially when the system is governed by a positive feedback loop, is proposed to be critical to coordinate bacterial group behaviors within a heterogeneous environment [32]. In addition to the examples discussed above, certain microbiota species affect *V*. *cholerae* virulence in an AI-2-dependent but LuxP-independent manner [33]. Upon attack by bacteria, mammalian cells produce a molecule that is structurally distinct but functionally similar to AI-2. In turn, this host-produced AI-2 mimic is detected by the bacterial LuxPQ and LsrB QS receptors [34]. Yet, the importance of this reciprocal interkingdom communication pathway in pathogenesis and symbiosis is not clearly defined. Moreover, *V*. *cholerae* possesses an additional QS circuit detecting an AI called 3,5-dimethyl-pyrazin-2-ol (DPO) with the receptor VqmA, separate from the multi-HK receptor pathway [35] (Fig 2). Interestingly, both DPO and AI-3 depend on TDH for biosynthesis, and DPO is a structural isomer of one of the compounds in the EHEC AI-3 family [10]. The VqmA/DPO system has been proposed as a bypass mechanism to optimize QS functions within specific niches, such as within the host. Whether the Vqm system also responds to host-derived signals remains to be studied.

What is the driving force for pathogenic and symbiotic bacteria to evolve to integrate both self-made AIs and host-derived chemical cues into their QS circuit? We envision that although the initial interactions between the host-derived signals and the QS receptors could be coincidental as some of these host-derived compounds structurally resemble the cognate self-made signal(s), one intriguing possibility is that these receptors might have evolved to serve as dual-function sensors to use the host-derived signal as a proxy for locating different regions in the animal host, as we have discussed in some of the *Vibrio* QS systems. Further investigation into the binding capacity and modulatory effects of host metabolites with other QS receptors present in different species could test this idea.
